# Fish (eggs) out of water: evolutionary divergence in terrestrial embryonic plasticity in Trinidadian killifish

**DOI:** 10.1098/rspb.2024.0083

**Published:** 2024-06-26

**Authors:** Matthew R. Walsh, Christopher Roden

**Affiliations:** ^1^ Department of Biology, University of Texas at Arlington, Arlington, TX 76019, USA

**Keywords:** phenotypic plasticity, amphibious, egg development, hatching plasticity, terrestrial incubation, desiccation

## Abstract

Externally laid eggs are often responsive to environmental cues; however, it is unclear how such plasticity evolves. In Trinidad, the killifish (*Anablepsoides hartii*) is found in communities with and without predators. Here, killifish inhabit shallower, ephemeral habitats in sites with predators. Such shifts may increase the exposure of eggs to air and lead to possible desiccation. We compared egg-hatching plasticity between communities by rearing eggs terrestrially on peat moss or in water. The timing of hatching did not differ between communities when eggs were reared in water. Eggs from sites with predators responded to terrestrial incubation by hatching significantly earlier compared with water-reared eggs. These responses were weaker in sites with no predators. Such divergent trends show that the presence of predators is associated with evolutionary shifts in hatching plasticity. Our results provide evidence for local adaptation in embryonic plasticity at the population scale.

## 1. Introduction

It has long been known that organisms exhibit the capacity to modify their traits in response to changes in environmental signals. This phenotypic plasticity is widespread across taxa and environmental stressors [1,2]. Research has also shown that eggs are responsive to environmental signals during development [3–6]. Egg developmental plasticity has been documented in a wide array of taxa including fish [7], reptiles [8], amphibians [9–12] and invertebrates [6,13] in response to stressors such as predators, pathogens, UV, desiccation and hypoxia. For instance, predator attacks induce faster rates of egg development and earlier hatching in red-eyed tree frogs (*Agalychnis callidryas*) [9]. Examples of embryonic plasticity that appear to be adaptive foreshadow that variation in environmental stressors has the potential to drive evolutionary changes in the responsiveness of eggs to the environment. In general, phenotypic plasticity is expected to evolve in spatially or temporally heterogeneous environments [14,15]. However, studies testing for evolutionary shifts in plasticity in egg development are lacking (but see [16]).

Many species of fish and amphibians lay eggs in terrestrial habitats that are intermittently exposed to air as they develop (reviewed in [3,5]). For instance, the mummichog (*Fundulus heteroclitus*) is a killifish that spawns in salt marshes during high tides by adhering its eggs to sea grasses [17,18]. These eggs are exposed to air during low-tide events and hatch upon re-immersion in water following predictable changes in tidal conditions. Plasticity in egg development and the timing of hatching is common in organisms that spawn in habitats where their eggs are exposed to air and may therefore desiccate. One strategy that has been documented is the ability to delay hatching upon air exposure (reviewed in [3,4]). Varela-Lasheras & Van Dooren [19] showed experimentally that the egg development rate of several species of non-annual killifish is slowed by exposure to air. Other studies have shown that some fish can accelerate rates of egg development upon experimental exposure to air [3,20].

*Anablepsoides hartii* is an amphibious species of killifish that is regularly observed outside water [21]. This species has been documented to emerge from water to avoid predators, aerially forage on terrestrial insects and disperse [22]. On the island of Trinidad, *A. hartii* (hereafter ‘killifish’) is found in communities that differ in the presence of predators [23,24]. This includes ‘high predation’ (HP) sites where they co-occur with several piscivorous predators (*Crenicichla frenata* and *Hoplias malabaricus*). In Trinidad, killifish are also observed upstream above barrier waterfalls in ‘killifish only’ (KO) sites, where they are the only fish species present. In HP sites, killifish experience increased mortality rates and are, in turn, found at lower densities with higher *per capita* resource availability [24,25]. Much research has shown that these ecological differences are associated with evolutionary changes in many phenotypic traits in killifish [24,26–29].

The presence of predators modifies the behaviour and habitat use of killifish in Trinidadian streams [30,31]. In HP sites, killifish are rare or absent from the open water of larger pools and instead occupy shallow, suboptimal and ephemeral habitats [30,31]. This includes shallow riffles, temporary side pools and rocky sections at the edge of streams, where the bodies of killifish are only partially submerged [30,31]. Furthermore, killifish lay eggs in terrestrial vegetation (leaves and roots) that is submerged in water at the edge of streams, and experiments have shown that they preferentially lay eggs in shallow water [25]. This is important because stream water levels are strongly influenced by precipitation that varies between wet and dry seasons each year. Therefore, any eggs laid in stream margins or side pools will potentially be exposed to air as water levels decline and such air-egg exposure will be increasingly probable during the dry season. More importantly, the shift towards shallower and ephemeral habitat in the presence of predators may, in turn, influence the frequency at which eggs are exposed to air and possible desiccation in HP sites. Such differences present the opportunity for divergent selection on embryonic plasticity.

Here, we tested for differences in egg-hatching plasticity in response to terrestrial incubation in killifish from HP and KO sites across two independent rivers. We reared eggs on the surface of moist peat moss or submerged in water. We then compared the timing of hatching and subsequent offspring performance (i.e. growth) between the fish communities. We had *a priori* expectations for how air exposure will influence hatching plasticity that is based upon the following background information: first, the volume of oxygen is higher on land versus in water and oxygen may diffuse faster across the egg membrane on land [32]. Second, similar to organisms that inhabit ephemeral habitats (i.e. annual fish and amphibians) [33], egg exposure to air represents a declining water level and, therefore, a habitat for killifish. Killifish eggs will rapidly desiccate under dry conditions but the likelihood that eggs will be re-submerged in water is unpredictable. Finally, killifish eggs need to be submerged in water to hatch. As a result, we expect that killifish eggs will respond to the terrestrial incubation treatment by hatching earlier versus eggs reared in water. If the influence of predators on the behaviour and habitat use of killifish leads to increased exposure of eggs to air and, in turn, modifies selection on egg-hatching plasticity, then we expect that the acceleration in the timing of hatching will be greater in HP fish.

## 2. Material and methods

These experiments used lab-acclimated, wild-caught killifish from HP and KO sites that were collected from two replicate streams (Arima and Aripo). All fish were collected using dip-nets in December 2022 or June 2022 and subsequently transferred to laboratory facilities at our university. Trinidadian killifish will naturally reproduce throughout the year. Individual rates of egg production in the wild are unknown but there is evidence that egg production is more pulsed in the presence of predators [25]. In the lab, killifish can reproduce daily. They typically lay small numbers of eggs at a time (1–3 eggs) but a single female was documented to lay > 50 eggs in a < 24 h period (Walsh, personal observation, 2024). For each population, we created mating pairings that consisted of one male and one female per 9 l aquaria. Each tank was supplied with habitat (polyvinyl chloride (PVC) pipes) and an artificial spawning substrate (plastic grass). The mating pairs were maintained under these conditions for 2–8 months prior to the initiation of the egg plasticity experiments. All individuals were fed a mixture of commercial flake food, brine shrimp or bloodworms daily. All experiments were performed in the same laboratory with a 12 : 12 (light:dark) photoperiod and temperature was maintained at 22°C.

We performed two separate experiments that manipulated the duration in which eggs were reared on peat moss. The treatments used in our follow-up experiment were based on the results of the first experiment. Our description of the methods, therefore, reflects the temporal sequence in which the experiments occurred.

### (a) 15 Day terrestrial incubation experiments

These experiments were initiated by collecting < 12 h old eggs from randomly assigned mating pairings. We placed one egg per well plate (24 wells per plate) and randomly allocated the eggs to one of the following treatments: (i) peat moss and (ii) water. The peat moss treatment consisted of eggs being reared on moist peat moss. The water treatment allowed eggs to develop in water. We added one drop of water daily to the peat moss to maintain a moist substrate. Methylene blue was added to all water used in these experiments to reduce the likelihood of fungal infections. Eggs were reared on peat moss for 15 days before they were transferred to water to initiate hatching on day 16. We used prior data on egg hatching dates to determine the number of days to rear eggs on peat moss. We assessed the timing of egg hatching in water from June to September 2023. The average day of hatching in water was 18.3 days ± 2.6 s.d. We transferred eggs from peat moss to water following day 15 because <5% of eggs in these previous data hatched by this date. This allowed ample time for eggs to develop on peat moss but the transfer occurred sufficiently early to determine if air exposure altered the timing of hatching. All eggs were monitored twice daily for hatching Monday-Friday (between 09.00 and 16.00) and once daily on weekend mornings (between 09.00 and 11.00). For these experiments, we collected eggs from 20 September 2023 to 6 November 2023. We used 7–9 unique families per population (no. of families per population: Arima HP = 7, Arima KO = 7, Aripo HP = 8 and Aripo KO = 9). The total sample number of eggs that ultimately hatched from each combination of population and treatment was: HP peat moss = 38, HP water = 23, KO peat moss = 40 and KO water = 38. A small number of eggs were originally allocated to the experiment, but they failed to develop and ultimately hatch (exposure to air = 4 eggs and water incubation = 4 eggs). We subsequently tracked rates of individual growth post-hatching. Newly hatched individuals were placed individually in small aquaria (~1 l) and were fed non-limiting quantities of brine shrimp nauplii twice daily. We assessed rates of growth by measuring each individual for wet weight after ~30 days (30–32 days post-hatching).

Variations in the timing of hatching and post-hatching growth were evaluated using general linear models in SPSS v. 27 (IBM Corporation). We included treatment (peat moss versus water), population (KO and HP) and river as fixed effects. We initially included all two-way interactions and the three-way treatment × population × river interactions in the analysis but removed any non-significant interactions and re-analysed the data without them. HP females were significantly larger than females from KO sites (*F*_1,135_ = 5.071, *p* = 0.026; total length in cm: HP = 5.87 ± 0.151 and KO: 5.43 ± 0.126). As a result, we included female total length (ln-transformed) as a covariate in the analysis for timing of hatching. We also had estimates of egg size for a subset of the eggs used in this experiment (101 eggs with images out of 139 total eggs used in the experiments). Egg size was quantified by taking an image of each egg upon initial collection and subsequently measuring egg size via ImageJ. Egg size did not differ between the populations and was therefore not included in the analyses (*F*_1,97_ = 1.11, *p* = 0.294; egg diameter in mm: HP = 2.18 ± 0.024 and KO: 2.15 ± 0.1018). In the analysis for post-hatching growth, 38 individuals were measured on day 31 or 32 (instead of day 30). We, therefore, included the age at which we assessed body size as a covariate but this effect was not significant and it was removed from the final model.

### (b) 12 Day terrestrial incubation experiments

Based on the results of the experiments described above (see Results), we performed a follow-up experiment that used a shorter incubation period on peat moss. Here, we used a 12 day terrestrial incubation to see if a shorter duration also led to observable differences in the timing of hatching between the fish populations (see Results). We used a 12 day treatment because that represents the earliest timing of hatching observed for eggs reared in water from our previous data. These experiments used the same lab-acclimated fish as the 15 day experiments, although we created new pairings at the start of the 12 day trials. These experiments included eggs from the following number of families per population: Arima HP = 5, Arima KO = 6, Aripo HP = 9 and Aripo KO = 6. The total sample sizes per populations were HP = 44 eggs and KO = 44 eggs. The protocols follow the methods described above. The key difference was that eggs were transferred from peat moss to water on day 12. We then tracked the timing of hatching daily. In this experiment, we did not include a treatment where eggs were reared entirely in water nor did we track post-hatching offspring growth. Differences in age at hatching were analysed using general linear models SPSS v. 27 with population, river and population × river interaction as fixed effects. We again included female body size as a covariate. The population × river interaction was not significant and was removed from the final model.

## 3. Results

### (a) 15 Day incubation experiments

#### (i) Egg hatching date

**Figure 1.**
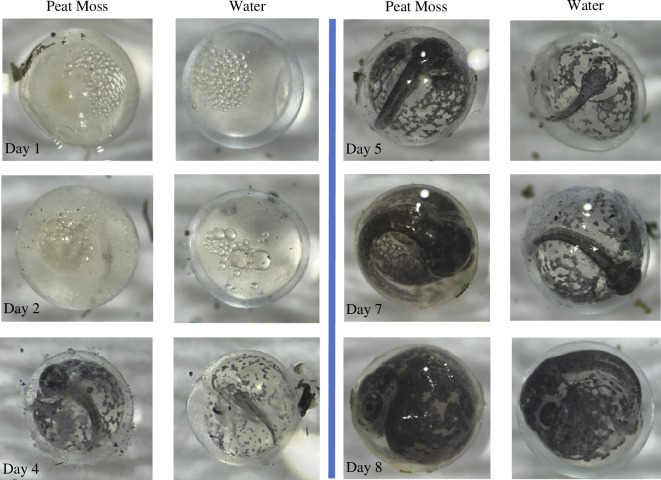
Sequence of development for eggs from high predation (HP) killifish. Each panel shows eggs that are the same age but were reared on peat moss or in water. The left side of the figure shows eggs on days 1, 2 and 4. The right side of the panel displays eggs on days 5, 7 and 8.

**Figure 2.**
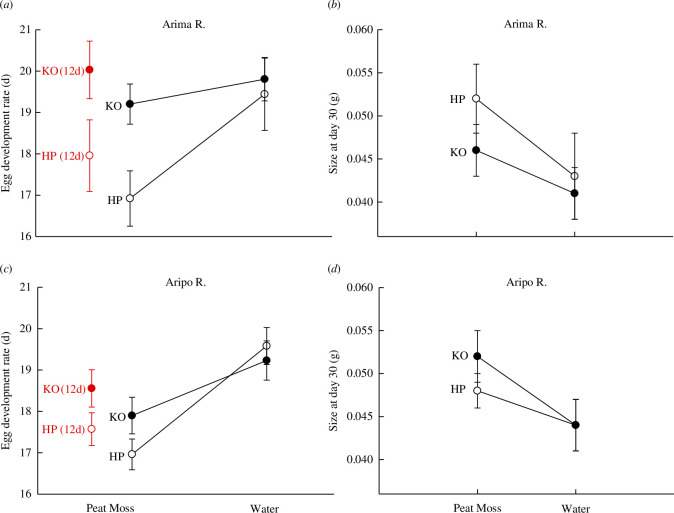
Influence of the 12 day and 15 day incubation treatments on (*a,c*) the timing of hatching and (*b,d*) offspring growth. Black closed circles—KO fish (15 day trials). Black open circles—HP fish (15 day trials). Red closed circles—KO fish (12 day trials). Red open circles—HP fish (12 day trials). Error = ±1.0 s.e. The left-hand panels are the fish from the Arima River while the right-hand panels include the fish from the Aripo River. The population × incubation treatment interaction was significant (*p* < 0.05) for timing of hatching. Error = ±1.0 s.e.

The effect of the incubation treatments on the timing of hatching differed between the fish communities; we observed a significant (*p* < 0.05) population × treatment interaction (table 1; figures 1 and 2. The differences in timing of hatching between HP and KO sites were minor when eggs were reared in water (figure 2). In the peat moss treatment, the eggs of HP fish exhibited a hatching date that was 9% earlier than the eggs of KO fish (figure 2). That is, the eggs of HP fish exhibited an acceleration in the timing of hatching in the peat moss treatment, but such a response was weaker or absent in the eggs of KO fish. The effects due to stream and population were not significant (*p* > 0.05; table 1). There was an overall significant effect of the incubation treatment (table 1; figure 2).

**Table 1.** Analyses of the terrestrial incubation experiments.

	15 day incubation					12 day incubation	
		hatching date		size at 30 days		hatching date	
	d.f.	*F*	*p*‐value	*F*	*p*‐value	*F*	*p*‐value
covariates							
female size	1	**6.97**	**0.009**			0.041	0.84
fixed effects							
stream	1	1.07	0.304	0.89	0.35	3.004	0.087
population	1	4.46	0.065	0.007	0.94	**5.52**	**0.021**
treatment	1	**25.15**	**<0.001**	**7.73**	**0.006**	…...	…...
population × treatment	1	**5.28**	**0.023**	…...	…...	…...	…...
error d.f.	133			127		83	

#### (ii) Post-hatching offspring growth

We observed an overall significant (*p* < 0.05) effect of the incubation treatment (table 1; figure 2). The mass of fish after ~30 days was 14% heavier when eggs were reared on peat moss compared with eggs that were reared in water. The effects due to population, stream and all statistical interactions were not significant (*p* > 0.05).

### (b) 12 Day incubation experiments

We observed significant differences in the timing of hatching between the populations (table 1; figure 2*a,b*). The eggs from HP females hatched 8% earlier than the eggs from KO females (figure 2*a,b*). The effects due to stream and the population × stream interaction were not significant.

## 4. Discussion

Our results clearly show that increases in predation are associated with phenotypic divergence in embryonic plasticity (figures 1 and 2). The eggs of HP fish responded to terrestrial incubation by hatching earlier compared with eggs that were reared entirely submerged in water (figure 2). Such responses to air exposure were weaker in the eggs of KO fish. These shifts in hatching plasticity were very consistent between replicate streams (figure 2). The differences in timing of hatching between the populations were similar when eggs were exposed to air for 15 days versus a shorter 12 day period (figure 2). We also found that post-hatching offspring growth was influenced by incubation conditions. Offspring that were reared on peat moss exhibited faster rates of growth than offspring whose eggs were reared in water, although such trends did not differ between HP and KO fish (figure 2). It is important to note that all females laid their eggs on submerged, floating artificial spawning substrates. As a result, the faster rate of hatching on peat moss in HP eggs was not influenced by maternal exposure to peat moss. Given the known influence of predators on the behaviour and habitat use of killifish in Trinidad [25,30,31], our results indicate that such shifts in habitat use and behaviour have driven evolutionary shifts in embryonic plasticity. While our experiments were performed using wild-caught individuals, all parents were acclimated to the lab for a minimum of 2 months (and upwards of 8 months) prior to the initiation of the experiments. This foreshadows that observed differences are probably genetic but additional common garden work using lineages that have been reared in the lab for multiple generations is needed to establish genetic differences in hatching plasticity between the fish communities. We also note that our conclusions apply to Trinidadian killifish (*A. hartii*) and we do not intend to extrapolate our results to all killifish.

Prior research has shown that eggs are sensitive to environmental conditions [34,35]. Similar to the present study, a significant amount of work has shown that the timing of development is altered by terrestrial incubation in fish and amphibians [3,5]. This includes studies showing that exposure to air can induce faster development in fish [20]. Other research has shown that exposure to predators [9,36–39], hypoxia [40,41], conspecifics [42] and disease [7] can alter the timing of hatching of eggs. Research has also shown that hatching plasticity can vary among closely related species [39,43]. Our results represent a key advance as they provide evidence for shifts in hatching plasticity among ecologically divergent natural populations (see also [16]).

The observed phenotypic divergence in the timing of hatching raises numerous questions about how and why such differences manifest. For instance, shifts in the timing of hatching can occur when all individuals hatch at the same stage of development, but earlier hatching occurs because of an increase in the rate of development [35]. The alternative is that earlier hatching occurs because individuals hatch at an earlier stage of development [35]. Furthermore, one reason that terrestrial incubation could lead to earlier hatching is because oxygen levels are higher (in terms of parts per thousand) in terrestrial versus aquatic environments and oxygen may diffuse more rapidly across membranes on land [32]. Oxygen availability is important because oxygen consumption increases as organisms convert yolk into metabolically active tissue. The differences in the timing of hatching between HP and KO sites on peat moss indicate that HP sites are better equipped to take advantage of increased oxygen in terrestrial environments but how and why they are able to do so is unknown. The underlying mechanisms that explain the differences in embryonic plasticity require further investigation.

To what extent are the patterns of hatching plasticity and the differences between HP and KO sites adaptive? In general, it is plausible that a faster rate of hatching in response to terrestrial incubation is potentially adaptive if it enhances survival and allows for an increased ability to hatch in response to unpredictable rain events. It may not be feasible for Trinidadian killifish to delay development (see [3,4]) if prolonged exposure to air increases egg/embryo mortality. This pattern of hatching may be more strongly favoured in HP sites if those fish experience increased episodes of air exposure and/or if increased predator-induced mortality simply favours maximum rates of development (see [24]). On the other hand, the patterns of hatching could be a physiological by-product of differences in oxygen volume and diffusion rates between terrestrial and aquatic conditions (see above).

## 5. Conclusions

We observed striking differences in egg-hatching plasticity between locally adapted populations of killifish (figure 1). Given the known impacts of predators on the behaviour and habitat use of killifish in the streams of Trinidad [25,30,31], our results provide new insights into how aquatic organisms adapt to increased exposure to terrestrial environments.

## Data Availability

All data have been archived in Dryad [44].
